# Verrucous carcinoma arising in an extended giant condyloma acuminatum (Buschke–Löwenstein tumor): a case report and review of the literature

**DOI:** 10.1186/1752-1947-7-273

**Published:** 2013-12-19

**Authors:** Mustapha Ahsaini, Yasser Tahiri, Mohammed Fadl Tazi, Jallaledine Elammari, Soufiane Mellas, Abdelhak Khallouk, Mohammed Jamal El Fassi, My Hassan Farih, Hind Elfatmi, Afaf Amarti, Roos E Stuurman-Wieringa

**Affiliations:** 1 Department of Urology, University Hospital Center Hassan II, 30000 Fez, Morocco; 2 Department of Pathology, University Hospital Center Hassan II, Fez, Morocco; 3 Department of Urology, Reinier de Graaf Gasthuis, P.O. box 5011, 2600, GA Delft, The Netherlands

**Keywords:** Buschke–Löwenstein tumor, Chemoradiotherapy, Condyloma acuminatum, Human papillomavirus, Verrucous carcinoma

## Abstract

**Introduction:**

Verrucous carcinoma of the external genitalia and perianal region is a rare variant of well-differentiated squamous cell carcinoma. It has been reported to have limited metastatic potential.

**Case presentation:**

We report the case of a 54 year-old Moroccan man who presented with locally advanced giant condyloma acuminatum (Buschke–Löwenstein tumor) after prolonged intervals of neglect (approximately 10 years). The disease covered his suprapubic, external genitalia and perianal region. It was locally aggressive with extensive tissue destruction. After a biopsy of the lesion, the diagnosis of verrucous carcinoma was confirmed. He initially received chemoradiotherapy, followed by extensive local excision, but he developed septic shock and died a few days later.

**Conclusions:**

The purpose of this case report is to present a case of verrucous carcinoma arising in an extensive giant condyloma acuminatum (Buschke–Löwenstein tumor) and discuss the literature on its diagnosis and management.

## Introduction

Verrucous carcinoma (VC) was first described as a distinct well-differentiated variety of squamous cell carcinoma (SCC) by Ackerman [1]. VC tends to appear mainly in oropharynx, genitalia and soles of the feet, although it may occur anywhere on the skin. Thus, VC has been known by several different names in relation to the anatomical site of the lesion. Different treatment modalities are described for patients with VC [2,3]. Buschke–Löwenstein tumor (BLT), or giant condyloma acuminatum (GCA), was first described by Buschke and Löwenstein in 1925 [4]. It is a rare sexually transmitted disease; the incidence is estimated to be 0.1% in the general population. It is characterized by invasive growth and recurrence after treatment, and malignant transformation is possible. VC resembles BLT in clinical appearance and histology. BLT is generally considered to be VC in genital regions, although there is still some confusion between BLT and VC; in some reports the lesions are regarded as distinct entities [5,6]. Moreover, the human papillomavirus (HPV) positivity rate in VC is lower than in common GCA [7,8]. To date, these questions remain unsolved, and several investigators have attempted to elucidate the etiology.

To the best of our knowledge, this is the first case report in which a history of BLT is complicated by malignant transformation covering a large part of the patient’s lower body. We present a recent systematic literature review regarding the clinical presentation and treatment of this rare tumor.

## Case presentation

**Figure 1:**
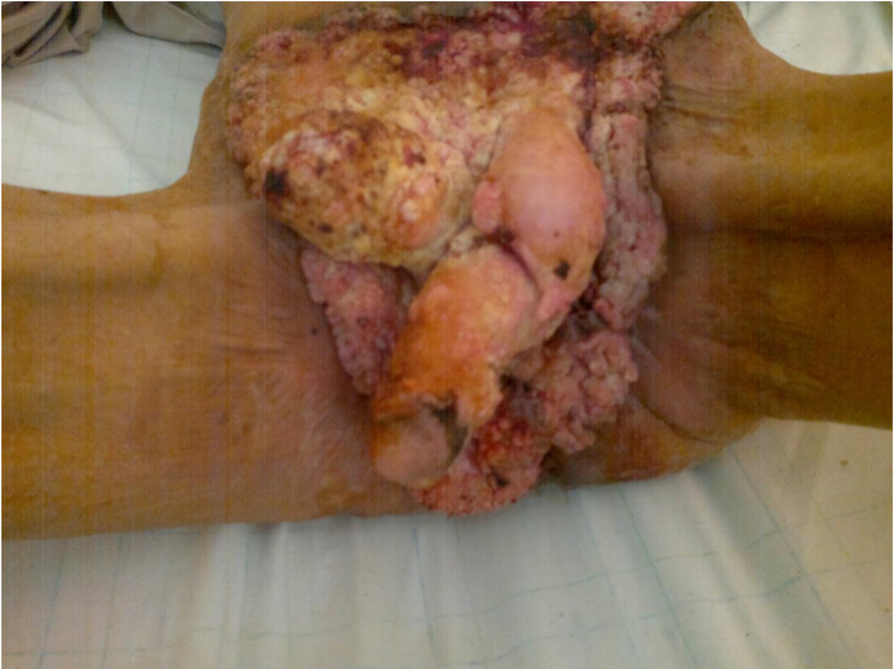
The tumor arising from the suprapubic, external genitalia and perianal region extended laterally to both thighs.

**Figure 2:**
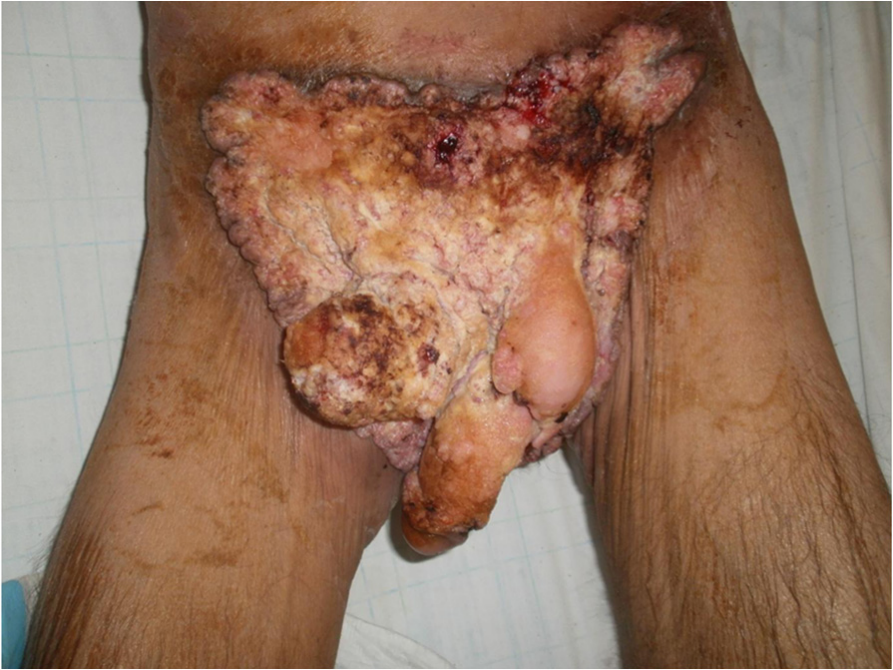
An extensive erosive, cauliflower-like growth involving a large part of the lower body.

**Figure 3:**
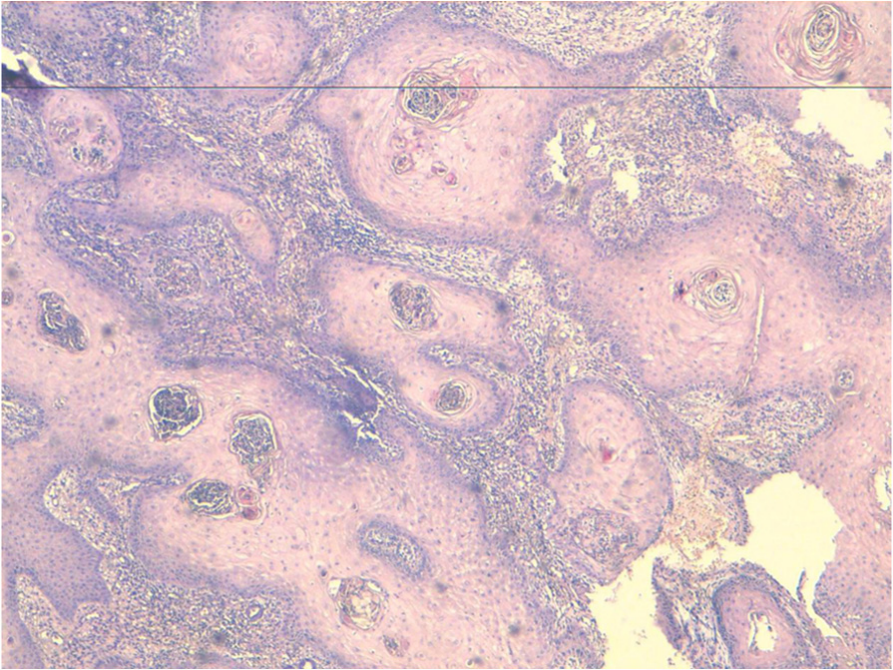
Verrucous architecture with papillomatosis, acanthosis and minimal loss of epithelial cell polarity (hematoxylin and eosin staining × 5).

**Figure 4:**
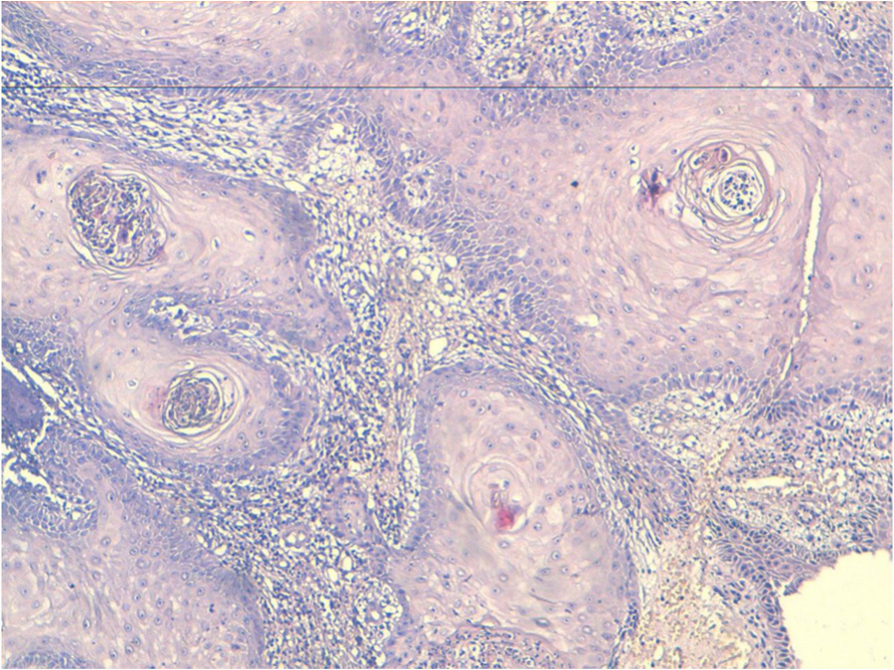
Koilocytosis and cellular binucleation (hematoxylin and eosin staining × 10).

A 54-year-old heterosexual married Moroccan man presented with a previous uncomplicated medical history. He did not report any risk factors for human immunodeficiency virus infection (HIV). He was seen in our emergency department with a foul smelling, exquisitely tender mass arising in his suprapubic, external genitalia and perianal region that extended laterally to both thighs (Figure 1). This lesion had been present for 10 years and had grown slowly over time. It had been increasing in size over the previous 8 months causing pain and bleeding. His delay was due to a hospital and needle phobia. A clinical examination revealed an extensive erosive, cauliflower-like growth involving a large part of his lower body (42 × 31cm in diameter; Figure 2). No inguinal or supraclavicular lymphadenopathy was detected clinically. He was admitted to the emergency room where multiple abscesses were drained and deep biopsies were taken from his tumor. The histological examination revealed a verrucous architecture with papillomatosis, acanthosis and a minimal loss of epithelial cell polarity (Figures 3 and 4). This confirmed the diagnosis of VC.

**Figure 5:**
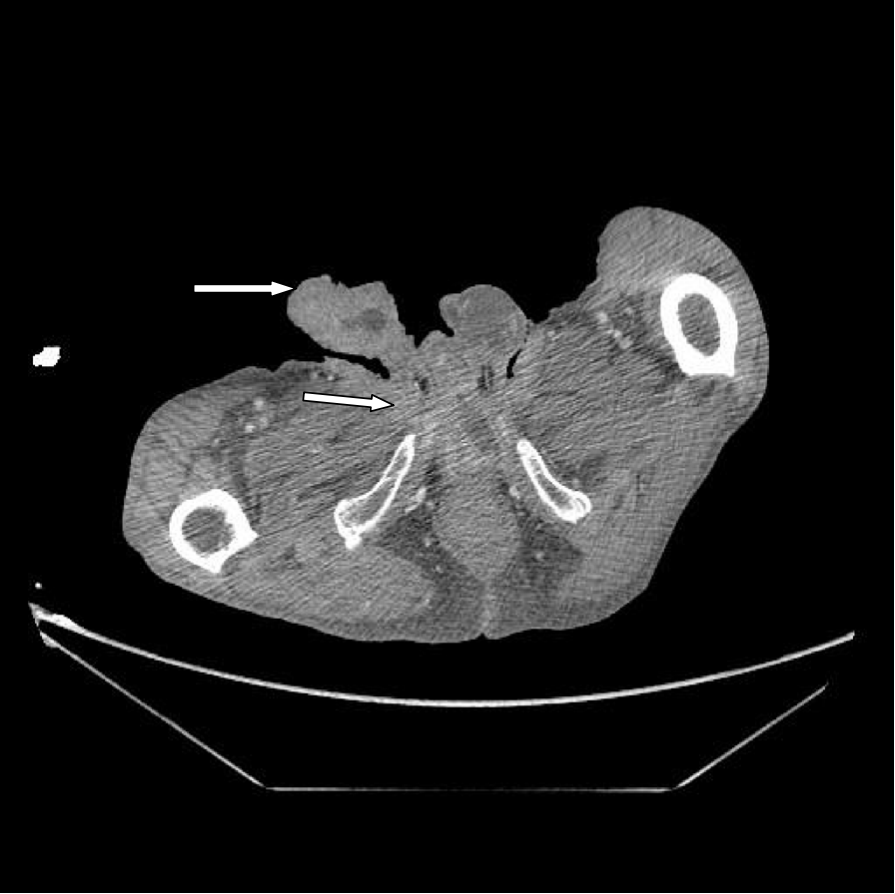
Computed tomography of the perineum showing the tumor spreading into the perineum and base of the scrotum (white arrows).

**Figure 6:**
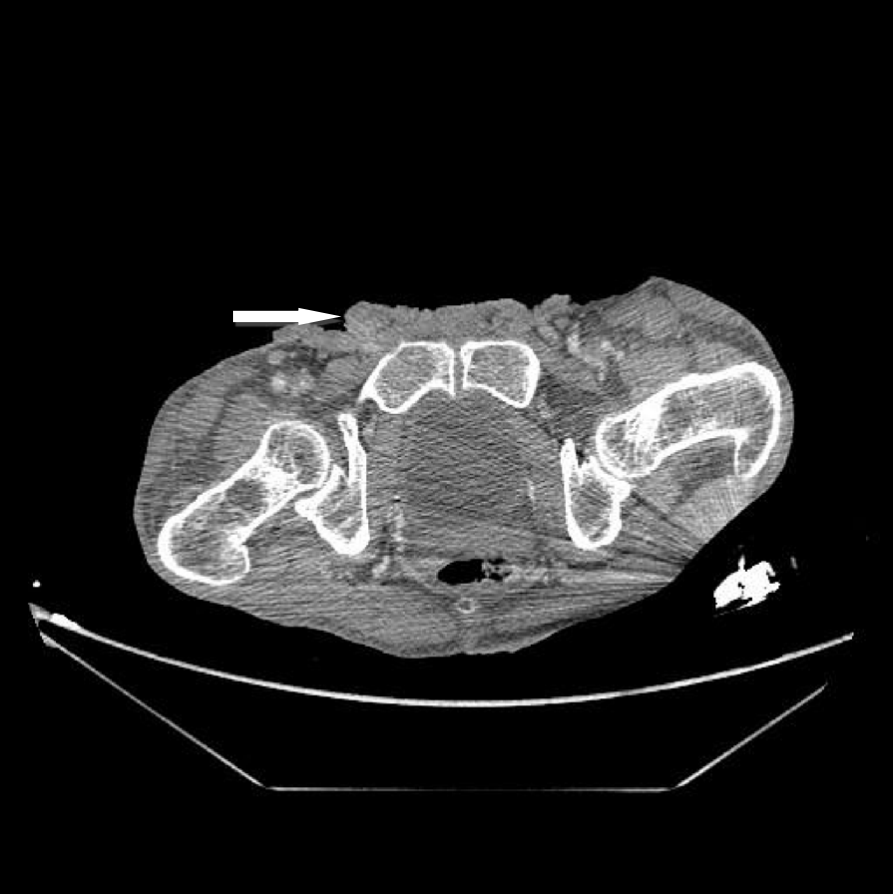
Computed tomography of the abdominal-pelvic floor showing the tumor invading the suprapubic region (white arrow).

Additional radiological investigations consisted of a thoracic-abdominal-pelvic computed tomography scan (Figures 5 and 6) which showed the localization of this tumor in the external genitalia, perineal and suprapubic region without any lymph nodes or distant metastases. The results of biochemical and serological investigations including a HIV test were normal. After discussion among the oncologist, radiotherapist, pathologist and surgeon, the patient was advised to undergo chemoradiation initially with the aim to reduce the size of his tumor followed by surgery.

After treatment of a local infection, our patient received two cycles of intravenous chemotherapy given three weekly with cisplatin 80mg/m^2^ on day one and 5-fluorouracil (5- FU) 600mg/m^2^ on day one to five given as a continuous infusion. After two cycles of chemotherapy the tumor size was reduced to 30% of initial size. The radiation was planned in two phases, two total doses of 45Gy in 25 fractions over 5 weeks, given at 1.8Gy per fraction. The combined therapy was well tolerated with just diarrhea as a side effect. Six months after completing the treatment, he underwent local excision with extensive abdominoperineal rectum excision and emasculation. In addition, a temporary diverting colostomy and a bilateral ureterostomy were constructed. Pedicled anterolateral thigh flaps and abdominal flap were used to cover the perineal defects. Unfortunately he developed septic shock and died some days later. The final pathology report after surgical resection of the tissue confirmed the diagnosis of VC.

## Discussion

BLT is a rare sexually transmitted disease triggered by HPV, usually genotype 6 or 11 [4]. Risk factors for HPV transmission are: multiple sexual partners, prostitution, homosexuality, lack of hygiene, and chronic genital infections. BLT is always preceded by condyloma acuminatum and the immune system is probably suppressed. It can be associated with congenital or acquired immunodeficiency, alcoholism, diabetes, or chemotherapy with immunosuppressive therapy [9]. Our patient did not have any of these risk factors. Other oncogenic subtypes, particularly HPV 16, 18, 31, and 33, have a well-known association with anogenital SCC [10]. Moreover, it is believed that malignant transformation could also be caused by the release of free oxygen radicals by activated inflammatory cells, inducing genetic damage and neoplastic transformation [11,12].

The absence of high-risk HPV subtypes in a case of BLT cannot exclude focally invasive SCC. Histopathological criteria for malignancy and clinical presentation continue to lead management decisions [5,6]. The average time for malignant transformation is known to be approximately 5 years [12]. Our patient had the tumor for approximately 10 years. The mean age of patients with BLT is 43 years, with a male to female ratio of 2:2. The risk of recurrence after excision is 60 to 66%, with an overall mortality of 20 to 30%. Malignant transformation has been reported in 30 to 56% of cases [12-14].

The disease is located on the penis in 81 to 94% of cases, in the anorectal area in 10 to 17%, and in the urethra in 5%. In females, the location is chiefly the vulva (90%) and an anorectal location is less frequent [15]. Suprapubic localization is rarely reported in the literature [16]. Our patient presented with an extensive lesion involving his suprapubic, external genitalia and perianal region that extended laterally to both thighs which is rarely reported in the literature.

Clinically, the tumor presents as exophytic fungating masses, sometimes with a cauliflower-like morphology. The gross appearance is generally a bulky tumor suggesting an aggressive behavior, whereas histopathology reveals a relatively low-grade malignancy. Biologically, this tumor shows a high recurrence rate and it is characterized by a low incidence of metastasis [17,18]. Malignant transformation may be suspected when bleeding, pain, and a rapid increase in tumor size [19] appears. Our patient had these complications in the last 8 months suggesting the malignant transformation was probably due to the time delay.

The diagnosis of VC requires evaluation of the clinical and microscopic appearance and biologic behavior of the neoplasm [20]. It is important to perform deep skin biopsies when clinical suggestion of VC exists.

VC presents as a distinct entity with an exo-endophytic growth pattern (in contrast to condyloma accuminata) of squamous cells showing mild atypia with pushing margins (in contrast to the invasive character of well-differentiated squamous carcinoma). This results in slow-growing lesions, locally invasive behavior, and very infrequent metastatic spread [11].

Differentiation between BLT and VC is difficult. Some authors consider these lesions to be similar. However, others maintain that BLT represents an intermediate lesion between condyloma acuminatum and VC, referring to it as a condyloma-like precancerous lesion [9].

Wide surgical excision, chemoradiation, topical and intralesional chemotherapy, carbon dioxide laser therapy, and photodynamic therapy have all been used in different combinations in the treatment of BLT, with varying success. Surgery is the treatment of choice and is effective in the early stages of the disease. Excision must be wide, and ranges from the Mohs technique when the size of the tumor is small, to abdominoperineal excision with pelvic lymph node dissection if lymph metastasis was suspected [14,21].

Radiotherapy alone is rarely used; it is usually used alone when excision cannot be performed or in recurrences. It may also be indicated to complement surgery in the case of an incomplete excision [14,21]. In addition, radiotherapy is controversial because of the potential for malignant transformation into aplastic carcinoma and metastasis [22].

Chemotherapy consists of a combination of cisplatin and 5-FU. The choice of this protocol was based on our own experience in treatment of patients with anal cancer. This combination has been used combined with radiation with acceptable toxicity and positives responses [23]. In patients with BLT with suspicion of malignant transformation, the optimal treatment is not well defined. Some authors recommend preoperative chemoradiotherapy to downstage extensive tumors, followed by radical surgery [24]. Tytherleigh *et al*. [25] reported the case of a patient with unresectable disease who received neoadjuvant chemoradiotherapy to downsize a tumor with subsequent complete surgical excision. In our patient, chemoradiotherapy allowed us to downsize the tumor (30%); wide excision of the tumor was made possible. However, due to this extensive procedure associated with significant morbidity and mortality, our patient died from septic shock despite extensive antibiotic treatment.

To avoid the recurrent disease some authors suggest the administration of an autogenous vaccine after surgical excision. The lowest reported recurrence rate at 1 year was less than 5% [26].

## Conclusions

VC is a rare, locally aggressive tumor. Surgical complete excision of VC is advised. Other treatment modalities such as chemotherapy or radiotherapy could be used to avoid mutilating surgical interventions. Based on our case and other published reports, chemoradiotherapy should be considered in patients with malignant transformation of GCA to downsize the tumor. Decision making in regarding to the goals of surgical intervention is complex and involves palliative excision versus a curative excision which unfortunately has the potential of significant morbidity and mortality. Wide surgical excision with local flap reconstruction can significantly improve the quality of life. A regular follow-up is necessary due to frequent recurrences and possible distant metastasis.

## Consent

Written informed consent was obtained from the patient’s next-of-kin for publication of this case report and any accompanying images. A copy of the written consent is available for review by the Editor-in-Chief of this journal.

## Competing interests

The authors declare that they have no competing interests.

## Authors’ contributions

MA was the principal author and major contributor in writing the manuscript. YT, MFT, SM, JE, AK, and HE analyzed and interpreted the patient data and reviewed the literature. RSW, MJE, MHF, and AA read and corrected the manuscript. All authors read and approved the final manuscript.
